# Genome sequence of *Planktotalea frisia* type strain (SH6-1^T^), a representative of the *Roseobacter* group isolated from the North Sea during a phytoplankton bloom

**DOI:** 10.1186/s40793-018-0311-5

**Published:** 2018-04-11

**Authors:** Insa Bakenhus, Sonja Voget, Anja Poehlein, Thorsten Brinkhoff, Rolf Daniel, Meinhard Simon

**Affiliations:** 1 Institute for Chemistry and Biology of the Marine Environment (ICBM), University of Oldenburg, Oldenburg, Germany; 2 Institute of Microbiology and Genetics, Genomic and Applied Microbiology and Göttingen Genomics Laboratory, University of Göttingen, Göttingen, Germany

**Keywords:** Marine bacterioplankton, *Rhodobacteraceae*, *Alphaproteobacteria*, *Roseobacter* group, Type IV secretion system, DMSP, Quorum sensing, Photoheterotrophy

## Abstract

*Planktotalea frisia* SH6-1^T^ Hahnke et al. (Int J Syst Evol Microbiol 62:1619–24, 2012) is a planktonic marine bacterium isolated during a phytoplankton bloom from the southern North Sea. It belongs to the *Roseobacter* group within the alphaproteobacterial family *Rhodobacteraceae*. Here we describe the draft genome sequence and annotation of the type strain SH6-1^T^. The genome comprises 4,106,736 bp and contains 4128 protein-coding and 38 RNA genes. The draft genome sequence provides evidence for at least three extrachromosomal elements, encodes genes for DMSP utilization, quorum sensing, photoheterotrophy and a type IV secretion system. This indicates not only adaptation to a free-living lifestyle of *P. frisia* but points also to interactions with prokaryotic or eukaryotic organisms.

## Introduction

The *Roseobacter* group features a global distribution in marine ecosystems like the water column and biological surfaces comprising up to 25% of marine microbial communities [1–3]. Members of this group exhibit numerous metabolic capabilities; besides aerobic anoxygenic photosynthesis and the production of bacteriochlorophyll *a*, they are also capable of oxidizing carbon monoxide, degrading aromatic compounds and catabolizing organic sulfur compounds [4]. Some representatives of this group are also able to synthesize secondary metabolites and to produce quorum sensing molecules like acylated homoserine lactones [5–7]. Genomic analysis showed that almost half of the marine *Roseobacter* genomes encode a type IV secretion system [4], thus, assuming to play a role in interactions of bacteria with other prokaryotic and eukaryotic cells including phytoplankton [8].

A recent study on genomic contents of the *Roseobacter* group identified a cluster of eight purely pelagic roseobacters which are distinct from the other members of this group [9]. One member of this cluster is strain HTCC2083, isolated from the coastal northwest Pacific Ocean [10]. *Planktotalea frisia*, the type species of the genus *Planktotalea* [11], is the closest relative of HTCC2083. *P. frisia* has been isolated from the southern North Sea, with highest abundances in spring and summer and constitutes up to 0.9% of the bacterioplankton [12].

In order to expand the knowledge on roseobacters prominent in marine pelagic systems we sequenced the genome of *P. frisia* and present the draft version together with its annotations. Even though SH6-1^T^ was originally allocated to the free-living fraction [13], experimental studies in which SH6-1^T^ was grown in presence of axenic algae cultures suggested specific interactions with different phytoplankton species. Furthermore, this representative of the *Roseobacter* group occurred mainly free-living during a phytoplankton bloom in the North Sea but also in the particle-associated fraction after the breakdown of a *Phaeocysti*s bloom [12]. Thus, our special focus was on genomic features related to the lifestyle of this organism and we had a closer look on genes involved in sulfur cycling such as degradation of dimethylsulfoniopropionate and genes indicating biofilm formation, motility, chemotaxis and quorum sensing pointing to a surface-attached lifestyle.

## Organism information

### Classification and features

**Fig. 1:**
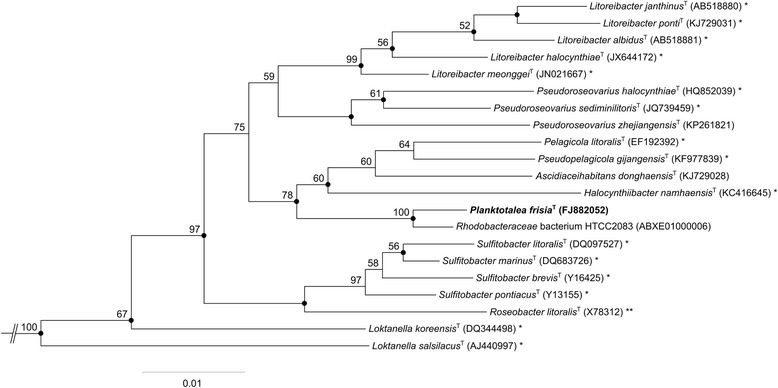
Phylogenetic tree highlighting the position of *Planktotalea frisia* strain SH6-1^T^ relative to other genome sequenced and type strains within the *Rhodobacteraceae*. The phylogeny was constructed with nearly full-length 16S rRNA gene sequences (> 1300 bp) using the neighbor joining tool of the ARB software [15]. The calculation of the tree also involves a bootstrapping process repeated 1000 times. Only bootstrap values ≥50% are shown. Filled circles indicate nodes also recovered reproducibly with maximum-likelihood (RAxML) calculation. Lineages with type strain genome sequencing projects registered in GOLD [16] are labeled with one asterisk, those listed as ‘Complete and Published’ with two asterisks [52]. Two sequences of *Staniera cyanoshaera* (AB039008, AF132931) were used as outgroup (not shown)

Figure 1 shows the phylogenetic neighborhood of *P. frisia* DSM 23709^T^ in a 16S rRNA gene sequence-based tree analyzed using NCBI-BLAST [14] and ARB [15]. The sequence of the single 16S rRNA gene copy in the genome does not differ from the previously published 16S rRNA gene sequence (FJ882052).

Strain SH6-1^T^ (= DSM 23709^T^ = LMG 25294^T^) was isolated from a water sample of the southern North Sea (54° 42’ N, 06° 48′ E) during a phytoplankton bloom from a water depth at 2 m in May 2007 [11].

**Fig. 2:**
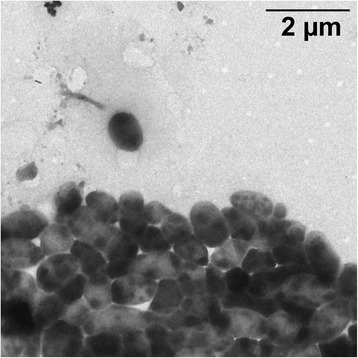
Transmission electron micrograph of *Planktotalea frisia* SH6-1^T^

Cells of *P. frisia* SH6-1^T^ are Gram-negative irregular rods with a width of 0.4 to 1 μm and a length of 0.5 to 4 μm (Fig. 2) [11]. On seawater agar colonies are small, circular, convex and whitish with a shiny surface. SH6-1^T^ is a marine, aerobic bacterium with a temperature range of 4–32 °C and an optimum growth rate at 20–25 °C. The salinity range for this strain is between 1.25 and 8% NaCl. The optimal pH range for growth is 7.5–9.0 with pH 6.0 being the lowest possible pH at which growth occurs under the tested conditions.

The following carbon sources were utilized by strain SH6-1^T^: L-alanine, L-arginine, L-aspartic acid, L-proline, L-serine, L-tryptophan, L-tyrosine, (+)-D-xylose, (+)-D-glucose, (+)-D-mannose, (+)-D-galactose, (−)-D-fructose, (−)-D-ribose, (−)-D-mannitol, sucrose, maltose, cellobiose, trehalose, lactose, sodium acetate, sodium pyruvate, sodium malate, citric acid, disodium succinate, sodium lactate, glycerol and Tween 80 [11]. Strain SH6-1^T^ cannot utilize L-asparagine, L-cysteine, L-glutamine, L-glutamic acid, glycine, L-histidine, L-isoleucine, L-leucine, L-lysine, L-methionine, L-phenylalanine, L-threonine, L-valine, (+)-L-arabinose, (+)-L-rhamnose, (−)-L-fucose, (−)-D-sorbitol, (+)-D-glucosamine, laminarin, starch, inulin, xylan, sodium formate, sodium propionate and DMSP [11]. Strain SH6-1^T^ is susceptible to penicillin G, streptomycin sulfate and chloramphenicol, but not to kanamycin sulfate. No growth was observed in the absence of the vitamins pantothenic acid and nicotinic acid amide [11]. A summary of the classification and features of strain SH6-1^T^ is presented in Table 1.

**Table 1:** Classification and general features of *Planktotalea frisia* SH6-1^T^ according to the MIGS recommendations [53] published by the Genome Standards Consortium [54]

MIGS ID	Property	Term	Evidence code^a^
	Classification	Domain *Bacteria*	TAS [55]
		Phylum *Proteobacteria*	TAS [56]
		Class *Alphaproteobacteria*	TAS [57, 58]
		Order *Rhodobacterales*	TAS [58, 59]
		Family *Rhodobacteraceae*	TAS [58, 60]
		Genus *Planktotalea*	TAS [11]
		Species *Planktotalea frisia*	TAS [11]
		Strain SH6-1^T^	
	Gram stain	Negative	TAS [11]
	Cell shape	Irregular	TAS [11]
	Motility	Slight motile	TAS [11]
	Sporulation	Not reported	NAS
	Temperature range	4–32 °C	TAS [11]
	Optimum temperature	20–25 °C	TAS [11]
	pH range; Optimum	6–9.5; 7.5–9	TAS [11]
	Carbon source	Amino acids, sugars	TAS [11]
MIGS-6	Habitat	Marine	TAS [11]
MIGS-6.3	Salinity	1.25–8% NaCl (*w*/*v*)	TAS [11]
MIGS-22	Oxygen requirement	Aerobic	TAS [11]
MIGS-15	Biotic relationship	Free-living	TAS [11]
MIGS-14	Pathogenicity	Not reported	NAS
MIGS-4	Geographic location	Southern North Sea	TAS [11]
MIGS-5	Sample collection	May 2007	TAS [11]
MIGS-4.1	Latitude	54°42’N	TAS [11]
MIGS-4.2	Longitude	06°48′E	TAS [11]
MIGS-4.3	Altitude	2 m below sea level	TAS [11]

^a^ Evidence codes - *TAS* Traceable Author Statement (i.e., a direct report exists in the literature), *NAS* Non-traceable Author Statement (i.e., not directly observed for the living, isolated sample, but based on a generally accepted property for the species, or anecdotal evidence). These evidence codes are from the Gene Ontology project [61]

#### Chemotaxonomic data

The principal cellular fatty acids of strain SH6-1^T^ are C_18:1ω7c_ (70.97%), C_18:2_ (11.45%), C_16:0_ (6.44%), 11-Methyl C_18:1ω7c_ (2.74%), C_12:1_ (2.56%), C_12:1_ 3-OH (1.82%), C_18:0_ (1.75%), C_10:0_ 3-OH (1.36%), C_14:1_ 3-OH (0.18%) and summed feature 7 consisted of C_19:1ω6c_ and/or unknown ECL 18.846 (0.34%) [11]. Ubiquinone Q10 is the predominant respiratory lipoquinone of strain SH6-1^T^ and the major polar lipids are phosphatidylcholine, phosphatidylglycerol, one unidentified aminolipid and one unidentified phospholipid [11].

## Genome sequencing information

### Genome project history

The genome was sequenced within the Collaborative Research Center “Ecology, Physiology and Molecular Biology of the *Roseobacter* clade: Towards a Systems Biology Understanding of a Globally Important Clade of Marine Bacteria” funded by Deutsche Forschungsgemeinschaft. The genome project was deposited in the Genomes OnLine Database [16] and in the Integrated Microbial Genomes database [17]. The Whole Genome Shotgun project has been deposited at DDBJ/ENA/GenBank under the accession number MLCB00000000. The version described here is version MLCB01000000. A summary of the project information is shown in Table 2.

**Table 2:** Project information

MIGS ID	Property	Term
MIGS-31	Finishing quality	Draft
MIGS-28	Libraries used	Nextera xt
MIGS-29	Sequencing platforms	Illumina GAiix
MIGS-31.2	Fold coverage	150×
MIGS-30	Assemblers	SPAdes v3.5
MIGS-32	Gene calling method	Prodigal v2.50
	Locus Tag	PFRI
	Genbank ID	MLCB00000000
	GenBank Date of Release	December 1, 2016
	GOLD ID	Ga0150920
	BIOPROJECT	PRJNA347625
MIGS-13	Source Material Identifier	DSM 23709^T^
	Project relevance	Tree of Life, environmental

### Growth conditions and genomic DNA preparation

A culture of SH6-1^T^ was grown in DSMZ medium 1282 (SH Seawater medium) [11] at 20 °C. Genomic DNA was isolated using a Power Soil DNA Isolation kit (MoBio) following the standard protocol provided by the manufacturer but modified by the addition of 100 μl Tris for cell lysis. DNA is available from DSMZ through DNA Bank Network [18].

### Genome sequencing and assembly

The draft genome sequence was generated using Illumina sequencing technology. For this genome, we constructed and sequenced an Illumina paired-end library with the Illumina Nextera XT library preparation kit and sequencing of the library using Genome Analyzer IIx were performed as described by the manufacturer (Illumina, San Diego, CA, USA). A total of 4.6 million paired-end reads were derived from sequencing and trimmed using Trimmomatic version 0.32 [19]. De novo assembly of all trimmed reads with SPAdes version 3.5.0 [20] resulted in 227 contigs and 150-fold coverage.

### Genome annotation

Genes were identified as part of the genome annotation pipeline of the Integrated Microbial Genomes (IMG-ER) platform using Prodigal v2.50 [21]. The predicted CDS were translated used to search the CDD, KEGG, UniProt, TIGRFam, Pfam and InterPro databases. These data sources were combined to assert a product description for each predicted protein. Non-coding genes and miscellaneous features were predicted using tRNAscan-SE [22], RNAmmer [23], Rfam [24], TMHMM [25] and SignalP [26]. Additional gene prediction analyses and functional annotation were performed within the IMG-ER platform [27].

## Genome properties

**Fig. 3:**
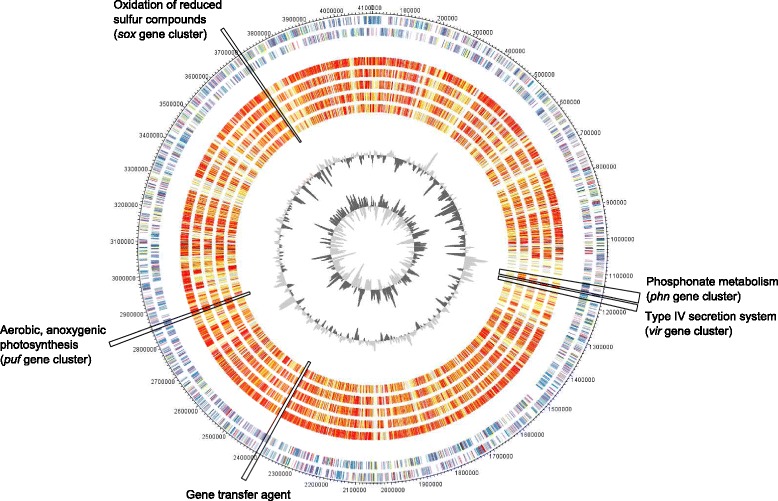
*Planktotalea frisia* SH6-1^T^ artificial circular chromosome map. Genome comparison of *P.frisia* SH6-1^T^ with 6 genome sequenced members of the Pelagic *Roseobacter* Cluster [9]. Circles (from outside to inside): 1 and 2: Genes encoded by the leading and lagging strand of *P.frisia* SH6-1^T^ marked in COG colors in the artificial chromosome map; 3–7: The presence of orthologous genes is indicated for the genomes of *Rhodobacterales* bacterium HTCC2083, *Planktomarina temperata* RCA23^T^, *Rhodobacteraceae* bacterium HIMB11, CHAB-I-5 SB2 and *Rhodobacteraceae* bacterium HTCC2255. The similarity of orthologous genes is illustrated in red to light yellow and singletons in grey (grey: >e^− 10^-1; light yellow: <e^− 50^- > e^− 10^; gold: <e^− 50^- > e^− 90^; light orange: <e^− 90^- > e^− 100^; orange: <e^− 100^- > e^− 120^; red: <e^− 120^-0). The two innermost circles represent the GC-content and the GC-skew

The genome consists of 227 contigs with a total length of 4,106,736 bp and a G + C content of 53.77% (Table 3). Of the 4166 genes predicted, 4128 were protein-coding genes, and 38 RNA genes. No pseudogenes or CRISPR elements were found. For the majority of the protein-coding genes (78.06%) a putative function could be assigned and the others were annotated as hypothetical proteins. The genome statistics are provided in Table 3 and Fig. 3. The distribution of genes into COGs functional categories is presented in Table 4.

**Table 3:** Genome statistics

Attribute	Value	% of total
Genome size (bp)	4,106,736	100.00
DNA coding (bp)	3,712,645	90.40
DNA G + C (bp)	2,208,074	53.77
DNA scaffolds	227	100.00
Total genes	4166	100.00
Protein coding genes	4128	99.09
RNA genes	38	0.91
Pseudo genes	0	0
Genes in internal clusters	975	23.40
Genes with function prediction	3252	78.06
Genes assigned to COGs	2877	69.06
Genes with Pfam domains	3425	82.21
Genes with signal peptides	349	8.38
Genes with transmembrane helices	871	20.91
CRISPR repeats	0	0

**Table 4:** Number of genes associated with general COG functional categories

Code	Value	%age	Description
J	204	6.26	Translation, ribosomal structure and biogenesis
A	0	0	RNA processing and modification
K	185	5.68	Transcription
L	106	3.25	Replication, recombination and repair
B	3	0.09	Chromatin structure and dynamics
D	32	0.98	Cell cycle control, Cell division, chromosome partitioning
V	54	1.66	Defense mechanisms
T	86	2.64	Signal transduction mechanisms
M	150	4.6	Cell wall/membrane biogenesis
N	17	0.52	Cell motility
U	36	1.1	Intracellular trafficking and secretion
O	164	5.03	Posttranslational modification, protein turnover, chaperones
C	228	7	Energy production and conversion
G	235	7.21	Carbohydrate transport and metabolism
E	420	12.89	Amino acid transport and metabolism
F	91	2.79	Nucleotide transport and metabolism
H	181	5.55	Coenzyme transport and metabolism
I	203	6.23	Lipid transport and metabolism
P	183	5.62	Inorganic ion transport and metabolism
Q	150	4.6	Secondary metabolites biosynthesis, transport and catabolism
R	320	9.82	General function prediction only
S	178	5.46	Function unknown
–	1289	30.94	Not in COGs

The total is based on the total number of protein coding genes in the genome

## Insights from the genome sequence

Genome sequencing of *Planktotalea frisia* SH6-1^T^ resulted in 227 contigs with sizes between 0.51 kb and 181 kb. A detailed view on plasmid organization was not possible due to the number and length of contigs of the draft genome, but scanning the genome for typical plasmid repABC-type replication modules from *Rhodobacterales* [28] resulted in three modules, suggesting that this strain carries at least three extrachromosomal elements.

Phage-mediated horizontal gene transfer is known to drive genomic diversity of bacteria and prophage-like structures are common in marine bacteria [29]. The genome of strain SH6-1^T^ carries a complete gene transfer agent cluster (PFRI_24010–24170) organized similar to the first genetically characterized GTA agent of *Rhodobacter capsulatus* RcGTA [30] containing 14 of the 15 genes but lacking the *orfg1* gene. RcGTA-like genes are present in all taxonomic orders of *Alphaproteobacteria* and within the *Roseobacter* group, except in most strains of the Pelagic *Roseobacter* Cluster, i.e. *Planktomarina temperata*, *Planktomicrobium forsetii*, *Rhodobacterales* bacterium HTCC2255 and HTCC2083 [3, 4, 9]. Only strain HTCC2150 of the PRC members encodes the GTA-like gene cluster [4].

Genes encoding type IV secretion systems (T4SSs), facilitating the transfer of proteins and nucleoprotein complexes by the formation of a pilus, were found in half of the analyzed genomes of marine representatives of the *Roseobacter* group [4, 8, 31]. Vir proteins are essential components for conjugation and hypothesized to play a role in the cell-cell contact between roseobacters and phytoplankton cells [8]. The T4SS seems to be a unique pattern of marine organisms within the *Roseobacter* group, some *Erythrobacteraceae* and *Caulobacteraceae* [32]. The genome of strain SH6-1^T^ also encodes the complete T4SS for translocating DNA or proteins into other cells. It includes the *virB* operon (*virB1* to − *11*, excluding *virB7*; PFRI_11620–11730) mediating the transmembrane channel formation and the *virD2* and *virD4* relaxase and coupling proteins (PFRI_35220, PFRI_35230) analogous to the archetypal *Agrobacterium tumefaciens* VirB/D4 system [33]. The presence of the Vir gene cluster in the genome of *P. frisia* indicates that this strain is able to transfer DNA and proteins into prokaryotic and/or eukaryotic cells.

Flagellar synthesis as well as motility seem to be of importance for surface attachment and biofilm formation in many *Proteobacteria* [34–36]. The genome of *P. frisia* SH6-1^T^ exhibits some genes for flagellar synthesis but covering only 8 of 30 analyzed COG flagellar families. Analysis of the corresponding genes revealed that the flagellar loci are located at the terminus of the single contigs as it is also the case for *Roseobacter* sp. strain MED193 with only 11 of 30 genes grouping into COG flagellar families [31]. Hence, a precise statement about the existence of a complete set and therefore a flagellum for strain SH6-1^T^ is not possible but should not be excluded due to the detection of slight wobbling under laboratory conditions [11]. The genome of strain *P. frisia* reveals, however, no genes encoding proteins associated to chemotaxis and the ability to move towards certain chemicals in the environment.

Roseobacters are well known to be involved in the transformation of dimethylsulfoniopropionate, a metabolite produced primarily by marine phytoplankton, either by demethylation or cleavage [4, 8, 37]. Strain SH6-1^T^ harbors genes for both, the cleavage and the demethylation pathway, indicating its ability to utilize DMSP. Two genes encoding for the dimethylsulfoniopropionate demethylase converting DMSP into methylmercaptopropionate [38, 39] are present but genes encoding the subsequent degradation of MMPA to acetaldehyde are absent from the draft genome sequence. Genes encoding for the alternative DMSP cleavage pathway are present in *P. frisia*, DddP (PFRI_00730), DddQ (PFRI_14360) and DddW (PFRI_38540) producing dimethylsulfide and acrylate, which is in contrast to previous studies where no DMS formation for *P. frisia* was detected [13].

Carbon monoxide can be an additional potential electron donor, which is formed by photolysis of dissolved organic matter. Only *Roseobacter* strains containing both the definitive form I and putative form II of the CO dehydrogenases large subunit (*coxL*) are capable of oxidizing CO under laboratory conditions [40]. *Planktotalea frisia* exhibits both gene structures the form I (*coxMSL*; PFRI_33480–33500) as well as form II (*coxSLM*; PFRI_01330–01350), but form I is lacking the downstream genes *coxDEF* detected in other genomes of the marine *Roseobacter* group [40]. Hence, it needs to be proved if this strain is able to use CO as an additional electron donor.

Inorganic sulfur compounds play an important role for mixotrophic growth in the marine environment with thiosulfate as common compound in seawater. The *Roseobacter* group makes use of the oxidation of thiosulfate to sulfate using the periplasmic Sox multienzyme complex like *Ruegeria pomeroyi* [41]. The genome of *P. frisia* SH6-1^T^ encodes proteins associated to a set of *sox* genes (*soxRSVWXYZABCDEF*; PFRI_19680, PFRI_14240, PFRI_37660–37740) suggesting that reduced sulfur compounds can be a complementary energy source.

The genome of strain SH6-1^T^ harbors genes for the high affinity phosphate transport system (*pstSCAB*; PFRI_11530–11560) and also for the transport (*phnCDE*; PFRI_11490–11510) and cleavage (*phnGHIJKLN*; PFRI_11290–11350) of phosphonate, a source of phosphorous (P) important when inorganic P becomes limiting [42].

Quite a few marine bacteria are capable of using light as an additional energy source. Proteorhodopsins are widely distributed in major bacterial groups like *Flavobacteriia*, *Alphaproteobacteria* and *Gammaproteobacteria* [43] and aerobic anoxygenic phototrophs are widely distributed within the *Roseobacter* group [2, 44] and also for *P. frisia* genes encoding subunits of the photosynthetic reactions center complex (*pufML*) were detected via specific PCR [13]. Genes for a functional photosynthetic gene cluster (PFRI_28770–28970, PFRI_19280–19350, PFRI_19150–19250) were found in the genome of SH6-1^T^. They include *bch* and *crt* genes coding for the bacteriochlorophyll and carotenoid biosynthetic pathways, *puf* genes coding for the subunits of the light harvesting complex and the reaction center complex, *hem* genes and also genes for sensor proteins. Due to the structure of the *puf*-operon and presence of the additional *pufX* gene, only reported for the anaerobic *Rhodobacter* lineage so far, *P. frisia* can be assigned to the phylogroup E according to Yutin et al. [45] occurring only in coastal oceans. In addition, two genes encoding blue light sensors using FAD (BLUF; PFRI_28190, PFRI_41660) are also present in the genome of strain SH6-1^T^ indicating possible blue light-dependent signal transduction.

To analyze the lifestyle of *P. frisia* the genome was also screened for genes associated with quorum sensing (QS). QS systems mediated by N-acyl-L-homoserine lactones (AHLs) provide significant benefits to the group and influence bacterial social traits such as virulence, motility and biofilm formation in many *Proteobacteria* including the *Roseobacter* group [46–49]. Genome analysis revealed the presence of genes encoding an N-acyl-L-homoserine lactone synthetase (*luxI* homolog; PFRI_23420) and a response regulator (*luxR* homolog; PFRI_23430) indicating that *P. frisia* can perform QS.

## Conclusions

In addition to biogeochemically important features reported previously from other sequenced strains of the *Roseobacter* group e.g. [3, 41, 50, 51], genome analysis of *P. frisia* SH6-1^T^, which is closely related to a member of the Pelagic *Roseobacter* Cluster [9], HTCC2083, revealed the presence of at least three extrachromosomal elements and genes associated with quorum sensing and type IV secretion systems.

Correspondingly, we assume that this strain can switch between free-living and an algal host associated lifestyle.

## Abbreviations

AHLs: Acyl homoserine lactones
DMSP: Dimethylsulfoniopropionate
IMG: Integrated microbial genomes
QS: Quorum sensing
T4SS: Type IV secretion system
